# Secondary antibody deficiencies: what’s around the corner?

**DOI:** 10.3389/fimmu.2025.1672413

**Published:** 2025-12-04

**Authors:** Gianluca Lagnese, Carla Messuri, Remo Poto, Gilda Varricchi, Giuseppe Spadaro

**Affiliations:** 1 Department of Translational Medical Sciences, University of Naples Federico II, Naples, Italy; 2 World Allergy Organization (WAO) Center of Excellence, Naples, Italy; 3 Center for Basic and Clinical Immunology Research (CISI), University of Naples Federico II, Naples, Italy; 4 Istituti Clinici Scientifici Maugeri-IRCCS Scientific Institute of Telese Terme, Benevento, Italy

**Keywords:** secondary, antibody, deficiency, immunology, hypogammaglobulinemia, hematological abnormality, algorithm decision

## Abstract

Secondary antibody deficiencies (SADs) are characterized by impaired humoral immunity, which can cause recurrent and severe infections. Several factors may contribute to SAD development, making it difficult to establish a clear etiological classification. This heterogeneity also leads to clinical variability, further complicating patient management and treatment strategies. Various diagnostic and therapeutic algorithms are often adapted from those used in primary antibody deficiencies, potentially resulting in under- or over-treatment. Key points include the decision to initiate Immunoglobulin Replacement Therapy (IgRT) and the duration of the treatment. Given the increasing prevalence of SADs and the limited availability of immunoglobulin products, it is important to clarify when IgRT should be started. In this review, we summarize and update the different etiologies of SADs and propose a diagnostic algorithm applicable regardless of the underlying cause. We also examine the possible treatment options and diagnostic tools that can assist in making the correct therapeutic choice.

## 1 Introduction

Secondary antibody deficiencies (SADs) are a group of diseases characterized by either a quantitative or qualitative alteration of humoral immunity. They are the result of various factors that interfere with the immune system functions (1), in wich, the significant reduction of IgG levels in serum (hypogammaglobulinemia), represent an important parameter (1). Hypogammaglobulinemia and poor response to vaccination are the most common features, leading to recurrent and/or severe infections of the upper and lower respiratory tract (2). In the last years, SADs have become more prevalent, with a frequency 30 times higher than primary antibody deficiencies (PADs). This rise is largely attributed to the increased incidence and survival rates of lymphoproliferative diseases as well as the frequent use of antineoplastic and immunosuppressive drugs targeting B lymphocytes (3). Despite the growing clinical relevance of SADs, there are currently no established working definitions and diagnostic criteria. Various underlying causes contribute to heterogeneity in clinical presentations and management strategies (4). Consequently, treatment approaches are often adapted from those used for inborn errors of immunity, particularly in PADs, including immunoglobulin replacement therapy (IgRT) (5, 6). IgRT involves the cyclic administration of polyvalent human IgG, which is derived from the plasma of thousands healthy donors. This preparation method ensures a diverse range of antibody specificities and physiological representation of different IgG subclasses (7). Since its introduction in 1952, IgRT has been a cornerstone in the treatment of primary immunodeficiencies and contributed to the reduction of infection-related mortality (8). Similarly, in patients with SADs, it is effective in reducing infectious complications. Nevertheless, not all patients require IgRT, and there are still uncertainties regarding when to initiate treatment, the appropriate dosage, and the duration of therapy (9, 10). In recent years, the global need for immunoglobulins has steadily increased. This rise is attributed both to a growing number of antibody deficiency patients and the expanded use as an immunomodulatory treatment in autoimmune, hematological, and neurological diseases. The COVID-19 pandemic highlighted the critical issues and vulnerabilities of a system that is becoming unsustainable (11). In response to these challenges, in Italy, the Agenzia Italiana del Farmaco (AIFA) released a guideline document in February 2022 to address the use of immunoglobulins in shortage conditions, prioritizing their administration in primary and secondary antibody deficiencies (12). As previously discussed, the clinical and etiological variability of SAD make it difficult to draft unique guidelines. This review aims to update a diagnostic and therapeutic algorithm for SAD, which can be used regardless of the specific cause.

## 2 Classification

An etiological classification of SAD is quite complex since multiple underlying causes may coexist in the same patient (3). Nevertheless, a primary division leads to the distinction into:

- Hematological immunodeficiencies, which are secondary to lymphoproliferative diseases and their treatments.
- Iatrogenic immunodeficiencies, resulting from medical interventions.
- Protein loss-immunodeficiencies, caused principally by nephrotic syndrome, chronic enteropathy, and lymphatic diseases.
- Immunodeficiencies due to other causes, such as infections or malnutrition.

The estimated prevalence of hypogammaglobulinemia within each entity is reported in **Table 1**.

**Table 1:** Estimated prevalence of hypogammaglobulinemia within each etiological entity.

Etiological factor	Prevalence of hypogammaglobulinemia	Influencing factors	Ref.
CLL	Up to 60%	Age, treatment, disease stage.	(13, 14)
MM	Up to 90%	Age, treatment, disease stage.	(15)
NHL	15-50%	Age, treatment, disease type and stage.	(16, 17)
Anti CD20	5-60%	Cumulative dose, clinical indication.	(18–20)
BTK inhibitors	N.A.	–	
Anti-CD38	Up to 100%	Dose, previous treatment.	(21–23)
CAR-T	30-40%	Previous treatment.	(24)
HSCT	>50%	Age, clinical indication, myeloablative treatment.	(25)
Glucocorticoids	0-60%	Dose, treatment duration.	(26)
Non biological immunosuppressant drugs	0-30%	Clinical indication, dose, treatment duration.	(27–29)
Belimumab	N.A.	Treatment duration, concomitant NS.	
Antiepileptic	0-18%	Treatment duration.	(30)
Clozapine	8%	Treatment duration	(31)
PLE	Up to 100%	Severity, Specific cause.	(32)
NS	8-58%	Severity, Treatment	(33, 34)
Skin disease	17%	Severity	(35)
Malnutrition	Near 0%	Severity	(36)
HIV	Near 0%	–	(37)
CMV, EBV	N.A.	Pre-existing immunodeficiency	
Measles	N.A.	Pre-existing immunodeficiency	

### 2.1 Hematological immunodeficiencies

Chronic Lymphocytic Leukemia (CLL), non-Hodgkin’s Lymphoma (NHL) and Multiple Myeloma (MM) are the most common causes of hematological immunodeficiencies. In these lymphoproliferative disorders, hypogammaglobulinemia can result from both the disease and its treatment, which typically involves medications that target B lymphocytes (38). Moreover, several additional factors may interfere with the T-cell compartment and innate immunity, further increasing the infectious risk in patients affected (39).

In CLL patients, antibody deficiency occurs in up to 60% of cases, depending on the patient’s age, disease stage and treatment (13, 14). The finding of reduction in serum Immunoglobulins before the diagnosis suggests the direct role of leukemic clone in determining hypogammaglobulinemia (40). Neoplastic cells suppress B cell maturation since the early stages and directly inhibit plasma cell activity (41). Although B cell dysfunction is the most visible aspect, it is generally associated with a deeper impairment of the immune system. Both the CD4+ and CD8+ T cells can be dysfunctional, since the normal cytokine pattern is disrupted (42). Even phagocytic and antigen-presenting cell activity is suppressed, while the complement system is generally defective (43, 44). There are still doubts about the prognostic role of hypogammaglobulinemia in CLL patients, but it is certainly associated with an increased risk of infection (45, 46). Moreover, the severity and frequency of infectious diseases are reduced after starting IgRT (47).

In MM, the main pathogenetic mechanism is the immunoparesis (48). Myeloma cells and the tumor microenvironment suppress the normal plasma cell development and function through a cytokine-driven mechanism (49). The result is the progressive reduction of polyvalent immunoglobulin and a loss of antigen specificity, with a consequent increase in susceptibility to infections (50). Moreover, myeloma cells downregulate other immune cells’ activity through the secretion of TGF-beta and IL-6 (51, 52). Neutrophils are quantitatively and qualitatively impaired through the overproduction of IL-10 and neutropenia can worsen after the introduction of several therapeutic agents, increasing the risk of bacterial infections (53, 54). Instead, the abnormal production of IL-17 and IL-21 leads to the perturbation of the interplay between plasma cells and T-Follicular Helper cells (55).

The prevalence of hypogammaglobulinemia in untreated NHL patients ranges from 15% to 50%, depending on the patient’s age and lymphoma type (16, 17). The exact mechanism by which NHL leads to hypogammaglobulinemia remains unclear. It is plausible that lymphoma cells directly interfere with the maturation or function of normal B lymphocytes (56). It cannot be excluded that the lymphoma is the first expression of a primary immunodeficiency (57). Besides the mechanisms leading to hypogammaglobulinemia, low levels of IgG at diagnosis are associated with a worse prognosis and a higher risk of hospitalization, regardless of lymphoma grade and stage (58, 59). The administration of chemo- and immunotherapy may worsen hypogammaglobulinemia and increases the infection rate, making IgRT mandatory (60).

### 2.2 Drug-related immunodeficiency

Anti-CD 20 antibodies are commonly used to treat CLL and NHL, either alone or in combination with other chemotherapeutic agents. Additionally, these monoclonal antibodies (mAbs) are utilized in the treatment of autoimmune diseases. Rituximab (RTX) is the first-in-class member and remains the most used after almost 30 years since its first approval (61). It’s a chimeric IgG1 antibody that targets the CD20 protein expressed by B lymphocytes, leading to cell death through antibody-mediated cytotoxicity, complement activation and antibody-mediated phagocytosis (62). In patients with autoimmune diseases, RTX administration generally slightly and transiently reduces serum immunoglobulin levels (63). Prolonged treatment and a higher cumulative dose do not seem to further reduce serum IgG levels (64). Even if cases of persistent and symptomatic hypogammaglobulinemia are described, generally immune reconstitution occurs 6–12 months after the withdrawal (65). Conversely, hypogammaglobulinemia may persist and worsen over the years in patients suffering from lymphoproliferative disorders (66). In B-cell NHL patients, post-RTX hypogammaglobulinemia appears in 30-40% of patients, with a peak of 60% in patients receiving RTX maintenance therapy (18). Several factors can explain this difference, such as the concomitant administration of chemotherapy and the preexisting compromission of the immune system. The family of anti-CD20 mAbs also includes Obinutuzumab, Ofatumumab, Ocrelizumab and Ublituximab (67). The effects of these newer drugs on the immune system have been studied in patients with multiple sclerosis since they are less used in lymphoproliferative diseases (68). In a recent meta-analysis, it was reported that 11% of patients tend to develop ypogammaglobulinemia during treatment. The highest risk was associated with the administration of Rituximab and Ocrelizumab. In most cases, however, it was a mild hypogammaglobulinemia, with serum IgG > 400 mg/dl (19, 69). No correlation with treatment duration and cumulative dose has been noted, and the only risk factors identified are patient’s age and pre-treatment IgG levels (70). The reason for the variable frequency of hypogammaglobulinemia associated with different anti-CD20 mAbs remains unclear. One possible factor could be the specific mechanisms of action, as the predominant B cell killing mechanisms may include complement-dependent cytotoxicity (Type I) or antibody-dependent cell-mediated cytotoxicity (Type II) (71). However, there are no significant differences observed in B cell depletion and repopulation rates between these two classes of antibodies. In contrast, B cell repopulation occurs more quickly with subcutaneous administration compared to intravenous administration, suggesting that pharmacokinetic characteristics may also play a role (20). The higher prevalence of SAD among Rituximab-treated patients may be related to its effects on T lymphocytes. It is unknown if these additional effects are shared with other drugs in this class, but Rituximab may increase Treg levels while reducing TH1 response, contributing to immune system impairment (72).

Bruton’s tyrosine kinase (BTK) inhibitors (BTKi) have been developed since 1999 and ibrutinib was the first in class drug, initially approved for treating relapsed/refractory Mantle cell Lymphoma (73). Nowadays it’s a milestone in CLL treatment, as first line therapy (74). The mechanism of action is the inhibition of BTK activity, which is fundamental for B-cell development and protection against apoptosis. Knowing the effects of inborn BTK mutations on the immune system, one would expect a high incidence of severe hypogammaglobulinemia in patients treated with BTKi (75). Nevertheless, in CLL patients, the administration of ibrutinib produce a partial humoral reconstitution after the first months of treatment (76). However, long term treatment is associated to serum IgG reduction and to an increased infection risk (77, 78). Generally, the effects of these drugs are reversible upon suspension, which however is only taken into consideration in cases of disease progression or severe toxicity (79).

CD38 is a regulatory protein expressed by lymphoid and myeloid cells (80) and targeted by Daratumumab, a fully human monoclonal antibody that induce complement-dependent cytotoxicity and antibody-dependent cell death (81). Due to the high expression of CD38 on plasma cells, daratumumab is approved for the treatment regimen for multiple myeloma (82). The possibility of directly targeting antibody-secreting cells has led to exploring the efficacy of this mAb in several autoimmune diseases (83). As expected, in MM patients, daratumumab administration further reduces the already impaired production of polyclonal IgG (21, 22), increasing their susceptibility to infections (23).

Bispecific antibodies (BiAbs) are engineered antibodies characterized by two different antigen binding domains, capable of recognizing two distinct epitopes (84). This characteristic allows to simultaneously target two receptors expressed by the same cell or to interfere with two mediators of the same pathway (85). However, the most innovative feature of bispecific antibodies used in oncohematology is to redirect the cytotoxic activity of T lymphocytes against tumor cells. Bispecific T-cell Engager (BiTE) antibodies activate T cells through binding CD3 and induce the formation of an immunological synapse with cells expressing the target antigen (86, 87). Blinatumomab couples T and CD19+B lymphocytes and represents the best therapy for refractory/relapsed B-cell Acute Lymphoblastic Leukemia (B-ALL) (88). The killing of B cells justifies the high rate of hypogammaglobulinemia secondary to Blinatumomab treatment (89). However, immunoglobulin reduction does not increase infectious risk and is usually reversible upon the suspension (90), hence IgRT is rarely necessary (91). Teclistamab and elranatamab target CD3/BCMA, while Talquetamab can bind CD3/GPRC5D: these three bispecific antibodies can be used as fourth-line therapy in patients with relapsing myeloma (92). B-Cell Maturation Antigen (BCMA) is a transmembrane receptor, preferentially expressed by mature B-lymphocytes and essential for the survival of plasma cells (93). Whereas CD38 and BCMA have a broader expression profile, G protein–coupled receptor class C group 5 member D (GPRC5D) is selectively located on plasma cells (94). Since all these three BiAbs target plasma cells, hypogammaglobulinemia is an expected consequence of their administration; however, IgG reduction is deeper in anti-BMCA-treated patients (95, 96). Different anti-CD20 and anti-CD30 bispecific antibodies, as well as trispecific antibodies, are recently or currently being studied in several B lymphoproliferative diseases, and hypogammaglobulinemia may be a potential adverse event (97–99).

Chimeric antigen receptor (CAR) T-cell therapy is a treatment modality that utilizes a patient’s own engineered T-lymphocytes. The synthesis and the incorporation of a surface receptor combining an antigen recognition domain with T-cell activation domains ensure the specific identification and elimination of cells expressing the targeted antigen (100). To date, six different CAR T-cell products have been approved, targeting either CD19 or BCMA expressed on the B cell surface (101). Therefore, they are licensed for the treatment of relapsed/refractory B cell malignancies, such as diffuse large B cell lymphoma, Mantle cell lymphoma, Follicular Lymphoma, MM and B-ALL (102). However, the innovative mechanism of action of this therapy might be exploited for the treatment of solid cancers and autoimmune diseases (103). In the hematological setting, the frequency and severity of hypogammaglobulinemia due to CAR T therapy are slightly variable, depending on the patient’s age, previous treatments and the specific product. According to clinical trial data, it occurs in up to 30-40% of patients, but the rate seems to be higher in real-life studies (24). The progressive reduction of serum Ig appears a few months after the treatment is administered and can persist for years. A late resumption of spontaneous Ig production is also described.

Hematopoietic Cell Transplantation (HSCT) is a treatment for both malignant and non-malignant blood disorders. HSCT can be classified into two types based on the source of the stem cells: autologous, which uses the patient’s own stem cells, and allogenic, which uses stem cells from a donor who is HLA-matched. The superior efficacy of allogenic HSCT in treating lymphoproliferative diseases may be limited by a higher mortality. However, advancements in conditioning regimens and improved management of graft-versus-host disease (GVHD) have led to better clinical outcomes and increased survival rates (104, 105). After the transplant, the engraftment of blood progenitors results in a gradual reconstitution of the immune system. Recovery of B-cells can take up to 24 months and is associated with transient impairment of humoral immunity. Since the development of memory B-cells may take as long as 5 years, post-transplant hypogammaglobulinemia is frequent (106). Several factors influence immunity recovery: the primary disease, the conditioning regimen, pre- and post-transplant therapies, GVHD occurrence and its treatment (25). Serum Ig levels, as well as the infection occurrence, need to be constantly monitored, and IgRT may be required in selected cases (107).

Glucocorticoids exhibit pleiotropic effects on the immune system, based on dosage and treatment duration (108). High-dose or chronic glucocorticoid treatment disrupts B cell development and immunoglobulin production (109), and the concomitant effects on T lymphocytes and innate immunity contribute to increasing risk of infectious diseases (110, 111). Reduction of serum immunoglobulin levels primarily affects IgG and may persist even after the withdrawal of steroid therapy in long-treated patients (26).

Non-biological immunosuppressant drugs are used primarily to treat autoimmune and inflammatory diseases, as well as to prevent chronic rejection in transplanted patients. Depending on the mode of action, these drugs may be classified as antimetabolite agents (methotrexate, mofetil mycophenolate, azathioprine), calcineurin inhibitors (Cyclosporin A, Tacrolimus), and mTor Inhibitors (sirolimus, everolimus) (112). After solid organ transplantation, hypogammaglobulinemia occurs in up to 30% of patients, with higher rates after kidney, lung, or heart transplant (27). In patients with autoimmune disorders and inflammatory bowel disease, hypogammaglobulinemia appears to be a less common complication. The high frequency of hypergammaglobulinemia before and the major control on protein loss after starting immunosuppressive therapy may explain the poor effects on serum IgG levels in the first year of treatment (28, 29, 113). However, it has been widely demonstrated that these drugs interfere with specific antibody production after vaccination, and the lack of data from long-term treatment may conceal possible complications (114, 115).

Janus Kinase (JAK)/signal transducer of activation (STAT) pathway is involved in the signaling pathways of numerous cytokines and growth factors, being essential in the early development of T- and B-lymphocytes. Therefore, loss of function mutations of the different isotypes of JAK are linked to various types of inborn errors of immunity (IEI) (116). Although JAK inhibitors have been used for over a decade, there is limited data on their impact on humoral immunity. It has been demonstrated that they modulate *in vitro* B cell activation and maturation and impair IgG production (117). However, they poorly interfere with post-vaccination antibody response *in vivo*, and hypogammaglobulinemia is rarely reported in patients (114).

on the risk of severe adverse events (SAEs) associated with their use. Studies have shown that JAK inhibitors can modulate B cell activation and maturation *in vitro*, which can impair IgG production. However, they do not significantly affect the recall humoral immune response. Consequently, these inhibitors have minimal impact on the antibody response following vaccination, and hypogammaglobulinemia is rarely reported in patients treated with them.

Belimumab is a fully human monoclonal antibody targeting B-cell Activating Factor (BAFF) and is licensed for the treatment of Systemic Lupus Erythematous (SLE) (118). BAFF plays an essential role in the development, survival and stimulation of B-lymphocytes through the binding with three TNF receptors: TACI (transmembrane activator and calcium-modulating cyclophilin ligand interacting protein), BCMA (B-cell maturation antigen) and BAFF-R (BAFF-receptor). Specifically, BAFF/BAFF-R interaction is crucial for the maintenance of follicular and marginal and plasma cells (119). Given the above, a high frequency of hypogammaglobulinemia would be expected in patients receiving anti-BAFF treatment, but clinical trials did not evidence clinically significant immunoglobulin depletion (120) and humoral response to vaccines seems to be preserved (121). However, long-term treatment is associated with a mild IgG reduction, coupled with a decrease in circulating B cells, especially in memory B cells and plasmablasts (122, 123). The combination of belimumab with other immunosuppressant drugs, as well as a higher disease activity score, is clearly associated to a significant increase in infection risk (124).

Two drug classes are unexpectedly associated to antibody deficiencies: anti-psychotics and anti-epileptics. The effects on the immune system elicited by anti-epileptic drugs have been known since 1971. Sorrell et al. reported a humoral response impairment, consisting in serum IgA decrease and poor antibody response to vaccination, in patients treated with phenytoin or carbamazepine (125, 126). The development of anticonvulsants-induced hypogammaglobulinemia is related to treatment duration and is reversible upon the suspension (30). Infectious risk is slightly increased, and Immunoglobulin administration is rarely required (127, 128). Among atypical antipsychotic drugs, clozapine has been extensively evaluated for its association to antibody deficiency. Similarly to antiepileptic drugs, the reduction in serum Ig levels is depending by drug exposure (31). The effects elicited on the immune cells have been recently elucidated: clozapine leads to the hyperphosphorylation of protein kinase B and the downregulation of ICOS trafficking (129, 130). The consequent development of hypogammaglobulinemia further increases the already high risk of respiratory infections and pneumonia among clozapine-treated patients (131). Given the difficulties in discontinuing the treatment, it’s necessary to evaluate the appropriate therapeutic strategies to lower infectious complications.

It’s important to remember that most of the therapeutic agents mentioned above may elicit additional effects that further suppress the immune response (see **Table 2**). Neutropenia represents one of the most frequent complications, contributes to increasing the risk of mycotic and bacterial infections, and should be properly managed independently of the IgRT administration (141).

**Table 2:** Additional effects on immunity and clinical implications elicited by the main agents responsible for secondary antibody deficiency (SAD).

Therapeutic agent	Effects on immunity	Frequency	Clinical implication	Ref.
Anti-CD20	Neutropenia	5-25%	↑ Bacterial and mycotic infections	(132–134)
	↓ CD8+ T cells	N.A.	↑ Viral infections	
BTK inhibitors	Impairment of neutrophil and macrophagic activity	NA	↑ Bacterial Infections ↑ M. Tubercolosis, A. Fumigatus, P. Jirovecii Infection	(135)
Anti-CD38	Neutropenia	>80%	↑ Bacterial and mycotic infections	(136, 137)
	↓ NK cells	100% (dose-depending)		
Anti-CD3/CD19	Neutropenia	20-40%	↑ Bacterial and mycotic infections	(138)
Anti-CD3/BCMA	Neutropenia	48-65%	↑ Bacterial and mycotic infections	(139)
Anti-CD3/GPRC5D				
CAR T	Neutropenia, Lymphodepletion	60-95%	↑ Bacterial and mycotic infections	(140)

### 2.3 Protein-losing immunodeficiencies

Hypogammaglobulinemia may appear in patients affected by protein-losing diseases. Since the pathogenic mechanism is the loss of serum protein, immunoglobulin reduction is generally associated with hypoalbuminemia, while B cell development and antibody production is not affected. For this reason, immune response following the vaccinations is often preserved and the infection rate is not increased.

Protein-losing enteropathies (PLEs) encompass more than 60 pathological conditions, characterized by an increased protein loss trough the gut lumen. The loss may be driven either by increased lymphatic pressure or by mucosal defects. Reduction in serum immunoglobulins involve mainly IgG and can be associated with a selective decrease in CD4+ lymphocytes, especially in lymphatic disorders (32, 142). Considering the common involvement of gastrointestinal tract among PAD, it’s important to accurately discriminate the two conditions. PLEs are characterized by high levels of fecal alpha1-anti-tripsin and by normal circulating switched memory B cells (143). Even if increased infectious susceptibility is not a common feature, opportunistic and recurrent infections are described among patients with intestinal lymphangiectasia (144, 145). Nevertheless, IgRT is rarely necessary, and therapeutic goal is the correct management of the primary disease.

Nephrotic syndrome (NS) is characterized by renal loss of serum proteins, generally due to glomerular involvement. Hypoalbuminemia is the main laboratory feature of NS, but protein loss also involves serum immunoglobulins, which are often decreased (146). The reduction, primarily affecting serum IgG and IgA, is related to the degree of albuminuria and is associated with increased susceptibility to infection. Moreover, the affected patients often require immunosuppressive treatment, leading to a further increase in infection risk (147, 148). Similarly to the others protein-losing immunodeficiencies, specific antibody titers following vaccination may be reduced according to the entity of IgG decrease. In contrast, specific IgG- and IgM-secreting memory B cell count is preserved, suggesting a normal immune competence in untreated patients (149). These findings confirm the efficacy of pneumococcal vaccination in reducing the infectious burden in NS patients (150). However, additional therapeutic interventions, including IgRT, may be necessary to prevent infectious complications, especially in patients treated with B-cell depleting treatments (151).

Less frequently, protein-losing immunodeficiencies can be caused by skin diseases, primarily severe atopic dermatitis and skin burns (35). The loss of skin integrity promotes not only the loss of serum proteins, including immunoglobulins, but also the entry of microorganisms, favoring infections (152).

### 2.4 Immunodeficiencies due to other causes

Malnutrition can affect immune system function and is widely associated with an increased risk of infections, especially in children. Both innate and adaptive immunity are variably compromised in malnourished individuals, depending on the type and the severity of nutritional deficit (153, 154). Even if hypogammaglobulinemia is not a common feature (36), several studies have demonstrated a poor antibody response following vaccination in children with an inadequate intake of essential micronutrients (155).

Viral infections can cause immune dysfunction through various mechanisms; however, this effect is more significant when microorganisms directly affect T- or B-cells. The paradigm, as well as the most common infection-related immunodeficiency, is certainly Human Immunodeficiency Virus (HIV) infection (4). The progressive loss of CD4+ T-lymphocytes and the role of these cells in coordinating immune response explain the severe immune impairments, leading to acquired immunodeficiency syndrome (AIDS). In addition to indirect mechanisms, HIV also has direct effects on B lymphocytes (156), resulting in reduced vaccine responses and a decline in memory B cells. These effects are more pronounced in untreated HIV patients and are partially reversed by anti-retroviral therapy (157, 158). Even if humoral immunity is mostly impaired, both directly and indirectly, HIV patients rarely develop hypogammaglobulinemia and usually develop hypergammaglobulinemia (37). Herpesviruses, particularly Cytomegalovirus (CMV) and Epstein-Barr Virus (EBV), can cause transient hypogammaglobulinemia (159). Persistent hypogammaglobulinemia following EBV infection may suggest an inherited immunodeficiency and should be carefully evaluated (160, 161). In both cases, however, it is always necessary to distinguish between an infection-induced nor a pre-existing hypogammaglobulinemia. Finally, the measles virus (MV) is another important cause of immunodeficiency. Although the virus primarily targets monocyte and macrophage cells, acute infection can affect B and T lymphocytes through killing, proliferation reduction and function impairment. The production of immunoglobulins may be temporarily decreased, but this usually does not require specific treatment (162).

## 3 Clinical management of SAD

In PAD patients, the most common warning signs are recurrent infections and the ever-increasing need for antibiotic therapy. More rarely, autoimmunity and lymphoproliferation may precede infectious diseases and even be the only clinical manifestations of the disease (163). The vague clinical presentation justifies the difficulties and the delay in diagnosis, with negative consequences on the prognosis and quality of life of patients (164, 165). The increased awareness of SAD and the knowledge of the possible causes favor a proper diagnosis, and the real question is when and how to treat hypogammaglobulinemia.

Clinical work-up should begin with a detailed and targeted clinical history focused on assessing infectious manifestations and researching the possible causes of antibody deficiency. It is important not to exclude *a priori* the diagnosis of primary immunodeficiency, especially in young patients, since lymphomas and autoimmune cytopenia requiring immunosuppressive treatment may be an initial presenting manifestation (166, 167). The increased susceptibility to bacterial infections, involving the respiratory and gastroenteric tracts, is typically associated with hypogammaglobulinemia, given the role of humoral immunity against capsulated microorganisms (168). Several definitions of recurrent respiratory infections have been proposed in children, but none in adult patients (169), so Jeoffrey Modell’s foundation definition is still the most considered (https://info4pi.org/library/educational-materials/). Infection frequency is not the only parameter to consider in assessing infectious risk; it should be combined with severity, hospitalization rates and duration (170). Viral and mycotic complications may underlie a deeper defect in cellular and innate immunity, requiring a different treatment strategy. In this context, it’s fundamental to take into consideration absolute neutrophil count: whereas the correction of neutropenia might prevent an inappropriate use of immunoglobulins, its persistence despite specific treatment might address to prompt IgRT initiation. The frequent involvement of the upper and lower airways favors the development of infection-related sequelae. Lung imaging and pulmonary function tests identify patients with bronchiectasis and/or Chronic Obstructive Pulmonary Disease (COPD) (171, 172). The presence of bronchiectasis is not only a surrogate marker of recurrent and severe infections, but it also favors bacterial colonization and superinfections, generating a vicious circle that is difficult to interrupt (173). Gastrointestinal involvement is less common than in primary immunodeficiencies but should not be underestimated. The appearance of prolonged diarrhea may be an expression of viral infections, while chronic diarrhea may be linked to intestinal amyloidosis in patients with MM. Regardless of the causes, diarrhea is an important cause of protein loss and may determine a further reduction in immunoglobulin levels: for this reason, gastrointestinal symptoms have to be always investigated. In patients with hematological immunodeficiencies, clinical assessment should include performance status evaluation. In patients affected by MM and NHL, Eastern Cooperative Oncology Group (ECOG) status is an independent predictor of infections and infection-related mortality. Similarly, the malignancy stage must be considered (174, 175).

The quantification of serum immunoglobulins should include IgG subclasses, and for a more accurate assessment of the deficit, it should be performed once a week for 4 weeks (176). Depending on IgG reduction, serum immunoglobulin levels should be monitored over time at variable intervals (177). In multiple myeloma patients, serum protein electrophoresis (SPE) is necessary to detect the M component, which may alter serum immunoglobulin levels. The addition of immunofixation electrophoresis (IFE) allows us to quantify the M-protein and establish polyclonal immunoglobulin levels (178), while serum-free light chain (sFLC) measurement is useful to identify non-secretory myeloma and in the diagnostic approach to hypogammaglobulinemia (179, 180).

The vaccine challenge test has always been considered fundamental to characterize the functioning of the immune system better. Impaired vaccine response is an important item according to the main diagnostic criteria for common variable immunodeficiency and is useful in deciding treatment strategy (181–183). Several vaccines can be useful for studying the humoral response *in vivo*; the most used are tetanus and diphtheria toxoid and polysaccharide pneumococcal vaccines. Protein vaccines (tetanus and diphtheria) elicit a T-dependent humoral response, while the antibody response following polysaccharide vaccines is T-independent. Since SAD patients may have a defect involving either both T and B cells or B cells alone, both types of vaccine can be helpful in diagnostic settings (184, 185). Nevertheless, there are conflicting opinions regarding the interpretation of this test (186). While most evidences agree on 4–6 weeks as the optimal time to measure post-vaccination antibody response, there are few certainties about what titer levels are considered adequate, as they are influenced by age, pre-vaccination titers and previous exposures to antigens (187–189). According to Bonilla et al, protective levels are set to 0.1-0.2 IU/ml for tetanus and diphtheria and 1.3 μg/ml for pneumococcal polysaccharide when IgG are measured through enzyme-linked immunosorbent assay (ELISA) (190). The discrepancy in results between the less expensive and more efficient multiplex methods and ELISA should lead to using the latter to prevent further complications in interpreting the results (191). Circulating B cell phenotyping and evaluation of memory B cells by flow cytometry may be helpful to overcome these limitations mentioned above.

During their development and maturation, circulating B cells are characterized by the expression of different membrane markers in addition to CD19 and CD20 (192). In healthy adults, up to 50% of circulating B lymphocytes are CD27^+^, a distinctive signature of memory B cells, and half of them are class-switched, expressing high-affinity IgG or IgA (193). This subset is generated during the germinal center reaction after somatic hypermutation and class-switch recombination processes, and their spawning goes together with plasmablast differentiation (194). Low levels of circulating switched memory B cells may indicate impaired plasmacell development and antibody production (195), either in primary antibody deficiencies or B-cell depleting therapies treated patients (196). As further confirmation, several studies confirmed a strong association between poor vaccinal response and reduced memory B cells, regardless of disease (197). On the other hand, the selective expression of CD38 by plasma cells accounts for the discrepancy between serum immunoglobulin levels and circulating memory B cells in daratumumab-treated patients and readily explains the rapid immune reconstitution following drug withdrawal (198).

## 4 Antibody deficiencies (SAD)

As mentioned above, a critical issue in the management of secondary antibody deficiency regards the correct treatment. The absence of unique guidelines has led to the proposal of several therapeutic algorithms adopted from PAD (5, 6, 170). The great variability in the underlying mechanisms makes it difficult to find a single therapeutic strategy for all forms of secondary immunodeficiency. The aim of the treatment is the reduction of infectious diseases and this objective may be achieved through one or more of these interventions:

- Antibiotic prophylaxis
- Vaccine administration
- Respiratory physiotherapy
- Immunoglobulin Replacement Therapy

See **Table 3**.

**Table 3:** Description of therapeutic options for secondary antibody deficiencies (SAD).

Intervention	Main indications	Advantages	Limitations	Clinical notes	Ref.
Antibiotic prophylaxis	Used in hematologic settings to prevent infections	Effective in reducing infections	Risk of antimicrobial resistance with prolonged use; limited evidence for long-term benefit; potential for adverse effects	Should be reserved for selected high-risk patients and not used indefinitely	(199–202)
Vaccine administration	Essential prophylaxis for patients with SAD	Variable, antibody-independent protection against infections	Live attenuated vaccines may be contraindicated; reduced antibody response in patients with B-cell depletion; vaccine efficacy may not be predictable based on antibody titers alone	Should be administered regardless of IgRT; monitor cellular response if needed	(203–207)
Respiratory physiotherapy	Improves lung function and reduces respiratory infections	Non-pharmacological support	Requires patient motivation and adherence; limited data on efficacy in SAD specifically; may not be sufficient as standalone intervention in severe immunodeficiency	Especially beneficial in patients with bronchiectasis or chronic lung disease	(208)
Immunoglobulin Replacement Therapy (IgRT)	Natural treatment for antibody deficiencies	Effective in reducing infectious complications	Limited global supply; potential side effects (e.g., infusion reactions, thromboembolic events); requires regular administration and monitoring; unclear duration in SAD compared to PAD	Dosage and route (IVIG vs SCIG) should be tailored; reassess need periodically	(5, 6, 9, 11, 209, 210)

Antibiotic prophylaxis has long been used in hematological settings, even before the availability of clear evidence regarding their efficacy (211). Recently, McQuilten et al. published the results of the RATIONAL trial. They compared the effectiveness of trimethoprim/sulfamethoxazole prophylaxis versus IgRT in patients with hematological secondary immunodeficiency, highlighting no significant differences in infection rates (202). A few years earlier, the TEAMM trial reported the effectiveness of daily 500 mg levofloxacin prophylaxis in newly diagnosed multiple myeloma patients. However, the short duration of the study raises concerns regarding the safety and the development of antibiotic resistance from prolonged use (199). Long-term prophylaxis with low-dose azithromycin has been evaluated in PAD patients, in addition to IgRT. The 2-year-long continuative treatment reduced respiratory exacerbations, without selecting macrolide-resistant microorganisms (200). However, the spreading increase in antimicrobial resistance should lead to an appropriate use of antibiotics (212). SAD patients are often characterized by an impaired immune system, chronic lung disease and frequent abuse of antibiotics, representing an at-risk population of multidrug resistance (201). For this reason, in addition to carefully selecting patients requiring antibiotic prophylaxis, this should not be considered a long-term treatment strategy.

Vaccinations are a fundamental prophylactic tool and should always be performed in SAD patients. The concomitant IgRT does not interfere with vaccine efficacy and does not replace the administration of seasonal anti-influenza and anti-pneumococcal vaccines (203). There are still doubts about the safety of live attenuated vaccines in this setting, so their administration is not advisable, similarly to PAD patients (204). Antibody production is only the “visible part” of the vaccine effects, and besides the role in diagnostics, it does not predict its effectiveness. The recent anti-SARS-CoV 2 vaccination campaign allowed us to better understand the effects of vaccination upon T cells and highlighted a variable and antibody-independent protection against the infection, depending on the cause of immunodeficiency (189, 205, 213). Although the activation of B cell and the subsequent production of neutralizing antibodies has always been considered as the key mechanism of vaccine-induced immunity, a growing number of evidence suggests a prominent role of T cells in conferring protection. Most of vaccines in routine use induce a “cellular response” which involves both CD4+ and CD8+ T cells and leads to the development of memory effector T cells (214–216). The difficulty in evaluation of this cytotoxic response does not allow the routinary use, but several evidence suggest its establishment in patients with B cell depletion and with a various grade of immunosuppression (188, 206). Finally, innate immune cells might play a paradoxical role in vaccine immunization trough the recently defined “trained immunity” (207).

Immunoglobulin replacement therapy represents the more “natural treating approach” in antibody deficiencies. The arguments about starting IgRT regard the drug’s safety, availability, and clinical efficacy in preventing infections. As previously mentioned, plasma-derived medical product availability is limited by plasma donations and the complexity of manufacturing procedures. Intravenous immunoglobulin (IVIG) preparations derive from pooled plasma of at least 1000 healthy donors, and up to 130 donations are needed to guarantee one year-long therapy for PID patients (217). The improvements in diagnostics and purification methods have almost eliminated infection transmission risks. Similarly, the rate of serious adverse events such as acute kidney injury or thrombotic events has strongly decreased (218). Anaphylaxis, generally due to the development of anti-IgA IgG or IgE, also reduced with the production of low IgA-containing products (219). Infusion-related adverse events, and specifically flu-like syndrome, remain the most common side effect of intravenous administration. They are related to patient’s characteristics and comorbidities and infusion rate; the risk is higher during the first infusions or after switching to another IVIG preparation (220). However, they are often self-limiting or, at most, require paracetamol or low-dose corticosteroid administration. Premedication regimens can prevent the onset of these side effects, as well as the subcutaneous administration (SCIG), which also ensures other benefits (221). The necessary steps to ensure the safety may require up to a year. As a consequence, immunoglobulin products may not contain specific antibodies against prone to mutation viruses or newer pathogens (222). Despite IgRT may confer protection against seasonal virus through cross-reactive antibodies, vaccination is always useful to enforce it (223). Several studies have evaluated IgRT in SAD patients, confirming its efficacy in reducing infectious complications (209, 224–226). Although the EMA provided clinical and laboratory indications for treatment, distinguishing patients who require IgRT from those who may benefit from other therapeutic options can be challenging (210).

**Figure 1:**
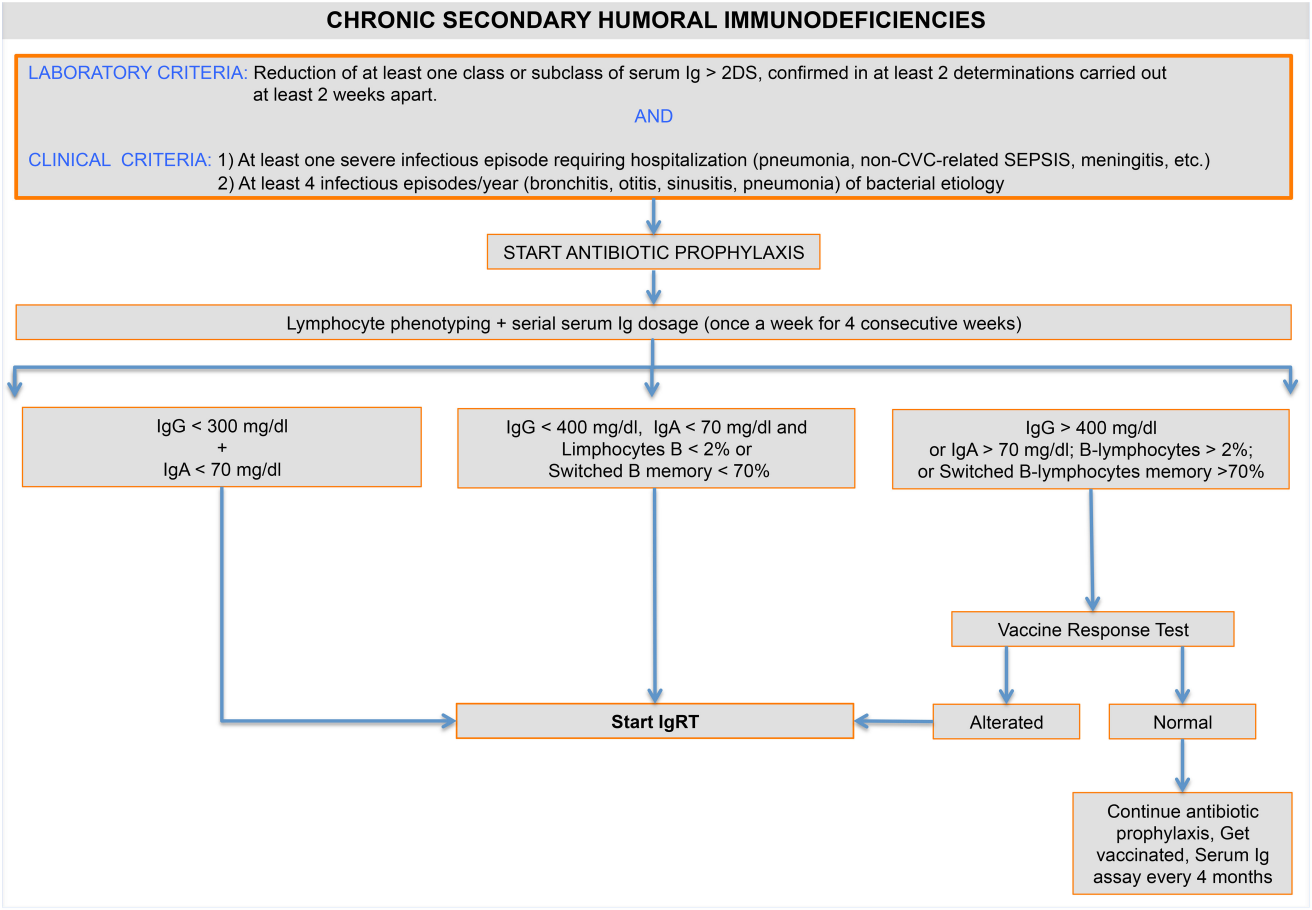
Chronic secondary humoral immunodeficiency. Diagram showing the criteria for SAD quantification and classification.

Clinical history, serum immunoglobulin values, and SAD-specific causes may assist in the decision to start IgRT. Ig testing should be repeated over 4 weeks to determine the minimum and maximum IgG levels. It is also important to evaluate the other isotype values, especially IgA. Lymphocyte and B-cell phenotyping should always be performed, while the vaccine challenge test is helpful in patients with recurrent infections and mild hypogammaglobulinemia. Conversely, antibody response to vaccination may be elicited by long-lived plasma cells, hiding a poor response to new antigens after B-cells depleting therapy (**Figure 1**).

Recurrent and/or severe bacterial infections are considered necessary for accessing IgRT. However, bronchiectasis and COPD favor infective superinfections and confer a higher infectious risk among hypogammaglobulinemic patients (172, 208, 227). For this reason, they should be evaluated in clinical assessment, and the presence of more than 2 exacerbations/year may be considered equivalent to recurrent respiratory infections. Similarly, the performance status score and the age in patients with hematological diseases identify more vulnerable patients. In drug-related immunodeficiency, the removal of the iatrogenic cause must always be recked, if possible. Nevertheless, aside from the feasibility difficulties, medication withdrawal does not ensure disease resolution. In symptomatic SAD patients, antibiotic prophylaxis may reduce infectious complications while awaiting laboratory test results.

Severe and persistent hypogammaglobulinemia (IgG < 300 mg/dl) associated with undetectable serum IgA (< 7 mg/dl) requires immediate immunoglobulin treatment, regardless of other laboratory findings, because of the higher infectious risk (170, 228). Protein-loss hypogammaglobulinemia represents the only exception: the primary intervention should aim at resolving the cause.

In patients with moderate Ig deficiency (IgG < 400 mg/dl and IgA < 70 mg/dl), IgRT may be started depending on B cell phenotyping. The reduction of circulating B lymphocytes (<2%) and/or switched memory B cells (< 70% of age-related normal values) indicates a failure in B cell development, suggesting the necessity of Ig administration (229) (**Figure 1**).

The vaccine challenge test is mandatory in patients with mildly reduced IgG levels, particularly those with normal serum IgA and B cell subsets. Despite the challenges in the interpretation, the 23-valent pneumococcal capsular polysaccharide vaccine is the most commonly used to evaluate T-cell independent humoral response (230). Patients with a significant reduction in specific antibody response following the vaccination may benefit of IgRT.

This approach is suitable for most of the SAD patients but may lead to an undertreatment of antibody deficiencies due to plasma cells depletion. Anti-CD38 and bispecific antibodies targeting BCMA or GPRC5D exert their effect downstream of B cell differentiation, directly compromising immunoglobulin production. In daratumumab-treated MM patients, poly-IgG levels rapidly decrease after treatment initiation, with a contextually increase in infection frequency. Lower levels of pre-treatment IgG, high-risk disease and concomitant/previous therapies are associated both to a more severe hypogammaglobulinemia and a higher infection rate (231). Interestingly, specific antibody response after vaccination is preserved, given the survival of low-CD38 expressing normal plasma cells (232). IgRT is effective in reducing infectious manifestations and should be started immediately afterward daratumumab initiation in patients with a higher infectious risk.

At the date, IgRT can be administered through two different routes: intravenous (IVIG) and subcutaneous (SCIG), while intramuscular administration has been definitively abandoned because of the serious local adverse events. Both IVIG and SCIG are effective in reducing infectious disease, but several studies highlighted some advantages of subcutaneous administration. It allows to achieve a steady state level, with higher and less variable serum IgG levels when compared to intravenous administration (233, 234). Furthermore, home-infusion improves patient’s quality of life and reduces the treatment-associated costs (235, 236). On the other hand, IVIG administration leads to a more rapid increase in IgG levels and for this reason it represents the best option for naive patients (237). The following switch to subcutaneous administration should be evaluated according to patient’s clinical conditions and preferences (238). Nevertheless, in patients with protein-loss associated diseases, SCIG therapy must be preferred (239).

**Figure 2:**
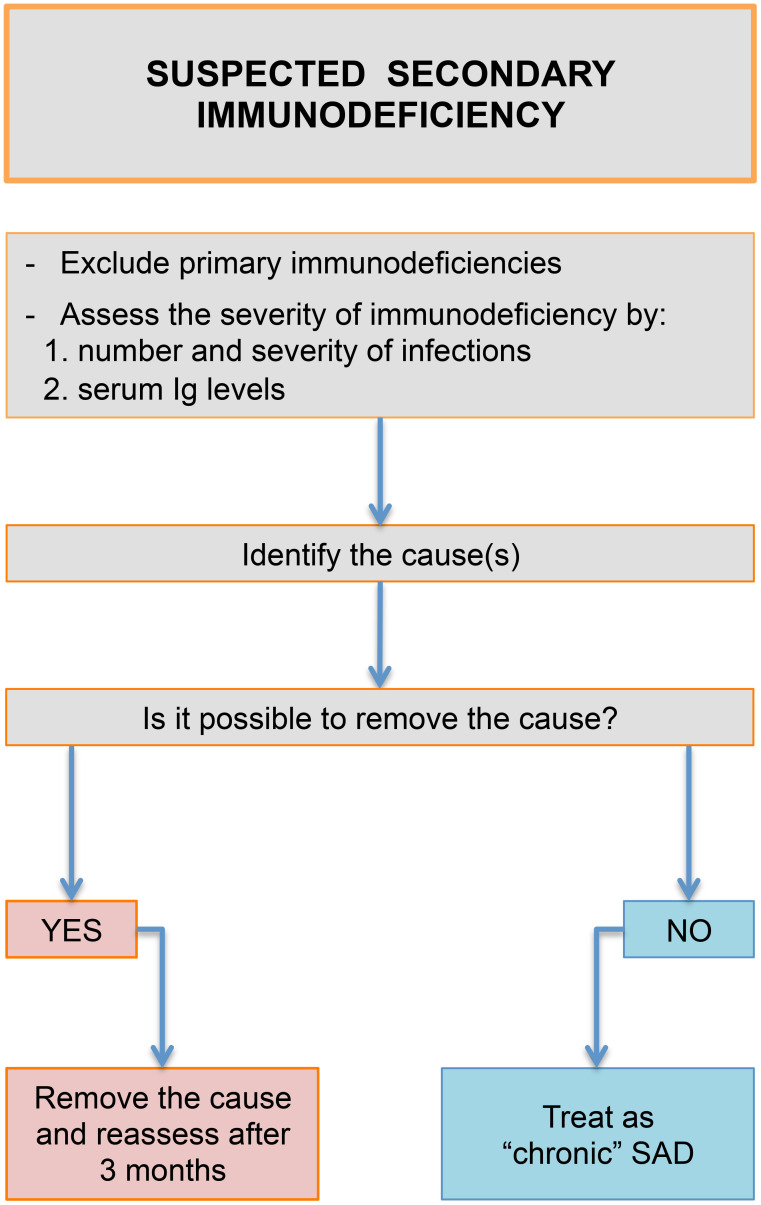
Secondary immunodeficiency. Decisional algorithm.

In PADs, especially in Common Variable Immunodeficiency, IgRT is a lifelong treatment. Generally, the proper monthly dosage is about 400–600 mg/kg but may be increased/decreased depending on trough level IgG values and infectious complications. The interval between administration differs with the route: it is approximately 3–4 weeks for IVIG, while in SCIG it can range from a few days (push therapy) to 2 weeks, reaching 4 weeks for facilitated-SCIG (fSCIG) (240). According to EMA indications, the authorized dosage of IgRT in secondary antibody deficiencies is similar to primary immunodeficiencies (241). However, real life studies suggested that a lower dosage is appropriate to guarantee an effective protection against infections (242). Moreover, there are few certainties about the treatment duration in this subset (**Figure 2**).

The increasing number of new diagnoses makes it necessary to think about IgRT discontinuation in selected cases (9). In MM patients, it might be discontinued after the suspension of plasma-cells depleting therapies and the reach of remission state (243). A similar approach may be deserved to patients treated with clozapine or immunosuppressant regimens. Different strategies should be adopted in patients treated with B-cell-depleting therapies or undergone allogenic HSCT. In these patients a B-Cell reconstitution is possible, even after years since IgRT starting (244, 245). Three factors can be evaluated to guide clinicians toward the withdrawal: infection rate, serum immunoglobulin levels and B-cells subpopulations (246, 247). The persistence of recurrent bacterial infections should postpone the decision about the interruption. A progressive increase of serum IgA and IgM may indicate a resumption in antibody production, since they are not altered by exogenous administration (248). Similarly, an increase in circulating total and switched memory B cells is suggestive of an immune reconstitution (249). This event is more frequent in patients with autoimmune diseases compared to lymphoproliferative diseases. However, treatment discontinuation should not be abrupt and should be achieved through a gradual reduction of the dosage until suspension. This approach enables to constantly assess infectious manifestations and to monitor serum immunoglobulin levels.

## 5 Conclusions

Secondary antibody deficiencies are an ever-expanding field, and their frequency is likely to further increase over the years. The progressive rise in immunoglobulin supply necessitates careful selection of patients who require IgRT. For this reason, clear indications regarding the proper diagnostic and therapeutic approach are essential. However, patient management cannot occur without a multidisciplinary approach, especially in patients with lymphoproliferative disorders. The involvement of onco-hematologists in the decision-making process is necessary, not only concerning the initiation of IgRT but also regarding eventual withdrawal.
